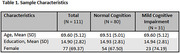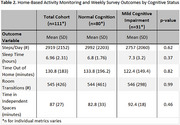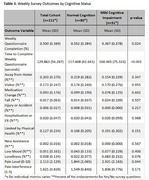# Digital health and survey response patterns among Hispanic/Latinx older adults with and without mild cognitive impairment: The COMPADRE‐CART study

**DOI:** 10.1002/alz70863_110537

**Published:** 2025-12-23

**Authors:** Marialy Salinas Valdez, Samantha Gates, Vanessa M. Young, Nathaniel Rodrigues, Wan‐Tai Michael Au‐Yeung, Kevin Cosgrove, Carlos A. Gaona, Layla Garcia, Roberto Garcia, Luis A. Mendez Rodriguez, Denisse Cisneros Garcia, Luis Humberto Serrano‐Rubio, Arash Salardini, Cynthia Cornejo, Ashley LaRoche, Lyndsey M. Miller, Sudha Seshadri, Jeffrey A Kaye, Tiffany F. Kautz, Mitzi M. Gonzales, Zachary T Beattie

**Affiliations:** ^1^ Glenn Biggs Institute for Alzheimer's and Neurodegenerative Diseases, University of Texas Health Science Center, San Antonio, TX USA; ^2^ Graduate School of Biomedical Sciences, University of Texas Health Science Center, San Antonio, TX USA; ^3^ School of Social and Behavioral Sciences, Arizona State University, Phoenix, AZ USA; ^4^ Glenn Biggs Institute for Alzheimer's & Neurodegenerative Diseases, University of Texas Health Science Center, San Antonio, TX USA; ^5^ Oregon Center for Aging & Technology (ORCATECH), Portland, OR USA; ^6^ Oregon Health and Science University, Portland, OR USA; ^7^ Texas Tech University Health Sciences Center, Lubbock, TX USA; ^8^ Department of Neurology, University of Texas Health Sciences Center, San Antonio, TX USA; ^9^ Glenn Biggs Institute for Alzheimer's and Neurodegenerative Diseases, University of Texas Health Science Center, San Antonio, TX USA; ^10^ Oregon Health & Science University, Portland, OR USA; ^11^ Boston University School of Medicine, Boston, MA USA; ^12^ The Framingham Heart Study, Framingham, MA USA; ^13^ NIA‐Layton Aging & Alzheimer's Disease Research Center, Portland, OR USA; ^14^ Cedars‐Sinai Medical Center, Los Angeles, CA USA; ^15^ NIA‐Layton Aging & Alzheimer's Disease Center, Portland, OR USA

## Abstract

**Background:**

Alzheimer's disease and related dementias (AD/ADRD) are a leading global public health concern, with an increased prevalence among underserved populations, including Hispanic/Latinx adults. However, cognitive and functional changes among Hispanic/Latinx adults remain understudied. COMPADRE‐CART is an ongoing longitudinal study that uses home‐based technology to detect health and activity changes among Hispanic/Latinx adults. Here, we examined differences in home‐based activity patterns and weekly digital survey completion metrics and responses between cognitively unimpaired (CU) adults and those with mild cognitive impairment (MCI) at baseline.

**Methods:**

Hispanic/Latinx participants, aged 62 years and older, were recruited from the South Texas catchment area. Participants completed the Uniform Data Set (UDS 3) clinical and cognitive assessments at baseline and received home‐based technology to continuously monitor their health and activity patterns. Room transitions, time spent outside the home, and time spent in independent spaces were evaluated using room sensors (NYCE). Steps per day and total sleep time were obtained from a wrist‐worn actigraph (Withings). Self‐reported mood and health changes were derived from weekly digital surveys (Qualtrics). Descriptive statistics were used to characterize activity and response patterns. Differences between the CU and MCI groups were evaluated using independent samples t‐tests.

**Results:**

Among 111 participants (*n* = 80 CU, *n* = 31 MCI, Table 1), we found no significant differences between groups in daily activity patterns (i.e., steps per day, sleep duration, time spent outside the home, room transitions, and time in independent spaces, *p* > 0.05, Table 2). However, participants with MCI showed lower weekly survey completion rates (36.7% vs. 55.2%, *p* = 0.024) and required longer time to complete the survey (168.6 vs. 117.7 seconds, *p* < 0.001) (Table 3). Additionally, those with MCI reported higher pain levels (3.0 vs 1.8 on a 0‐10 scale, *p* = 0.044), though self‐reported pain interference with daily activities did not differ significantly. Other self‐reported health events, including falls, hospitalizations, mood changes, and loneliness, showed similar frequencies across both groups.

**Conclusion:**

Weekly digital questionnaire metrics show promise in detecting subtle functional differences between MCI and CU Hispanic/Latinx adults. Longitudinal monitoring may provide more insight into whether these differences predict cognitive decline over time.